# Development of antimicrobial gelatin films with boron derivatives

**DOI:** 10.3906/biy-1807-181

**Published:** 2019-02-07

**Authors:** Sanem ARGIN, Merve GÜLERİM, Fikrettin ŞAHİN

**Affiliations:** 1 Department of Food Engineering, Faculty of Engineering, Yeditepe University , İstanbul , Turkey; 2 Department of Genetics and Bioengineering, Faculty of Engineering, Yeditepe University , İstanbul , Turkey

**Keywords:** Gelatin films, antimicrobial packaging, boron, antifungal, boric acid, disodium octaborate tetrahydrate, sodium pentaborate

## Abstract

Food packaging technology has been advancing to provide safe and high quality food products and to minimize food waste. Moreover, there is a dire need to replace plastic materials in order to reduce environmental pollution. The aim of this study was to prepare biodegradable antimicrobial packaging films from gelatin. Boric acid, disodium octaborate tetrahydrate, and sodium pentaborate were incorporated as the antimicrobial agents. Films containing boric acid and its salts showed antibacterial effect against Staphylococcus aureus and Pseudomonas aeruginosa, as well as antifungal and anticandidal effects against Aspergillus niger and Candida albicans. The mechanical strength of the films was mostly enhanced by the addition of boron derivatives. The rheological measurements and Fourier-transform infrared spectroscopy results suggest that boron derivatives did not interfere with the network formation during gelling. The morphology of boron-added antimicrobial films was found to be similar to the morphology of the control . In conclusion, the newly developed gelatin films containing 10% or 15% disodium octaborate (g/g gelatin) might be good candidates for biodegradable antimicrobial packaging materials.

## 1. Introduction

Petroleum-based plastics are commonly preferred in food packaging due to their low cost, ease of handling, various production technologies, lightweight, good barrier properties, and transparency (Shah et al., 2008; La garon and Lopez-Rubio, 2011 ). However, the risks of plastics to human health (Murphy et al., 1992; Date et al., 2002; Choi et al., 2005; Bošnir et al., 2007; Khaksar and Ghazi-Khansari, 2009; Pinto and Reali, 2009; Bach et al., 2012) and their adverse effects on the environment are well known (Shah et al., 2008; Gomez-Estaca et al., 2009; Cerqueira et al., 2011) . Given that the food industry is the largest industry where plastic packaging materials are used (Lagaron and LopezRubio, 2009) , there is a dire need to develop packaging materials that are biodegradable, renewable, and environmentally friendly (aThranathan, 2003; Jost and Stramm, 2016) . Composed of polymers that can be degraded by microorganisms (Nur Hanani et al., 2014) , biodegradable films made of proteins generally exhibit better mechanical and barrier properties than those made of polysaccharides (Cuq et al., 1998; Wang et al., 2007) . Gelatin, an animalbased protein, has been widely used in biodegradable food packaging and edible film studies (Sobral et al., 2001; Bigi et al., 2004; Chambi and Grosso, 2006; Andreuccetti et al., 2009; Rivero et al., 2010; Nur Hanani et al., 2012a, 2014; Li et al., 2014) . Gelatin is derived from collagen by treatment with acid (Type-A gelatin; isoelectric point is 6.0–9.0) or base (Type-B gelatin; isoelectric point is 4.8–5.1) (Djagny et al., 2001; Schrieber and Gareis, 2007; Gómez-Guillén et al., 2011) . Gelatin easily forms thermoreversible gels when cooled below 30 °C.

To date, different agents have been added to gelatin films to develop antimicrobial packaging materials and edible films (Gomez-Estaca et al., 2009; Pereda et al., 2011; Ahmad et al., 2012; Nowzari et al., 2013; Arfat et al., 2014; Kanmani and Rhim, 2014a, 2014b; Cozmuta et al., 2015; Martucci et al., 2015; Shankar et al., 2015; Clarke et al., 2016) . Although many studies have shown the antimicrobial properties of boric acid and boron derivatives (Bailey et al., 1980; Houlsby et al., 1986; Qin et al., 2010; Bursali et al., 2011; Dembitsky et al., 2011; Saita et al., 2012; Yilmaz, 2012; Sayin et al., 2016) , there has been no study incorporating boron compounds into food packaging materials. Boron addition to the packaging materials can be considered safe since boron is a part of the daily human diet (Rainey et al., 2016; Kuru et al., 2018) . Moreover, boron compounds are readily consumed as dietary supplements due to their therapeutic effects (Korkmaz et al., 2014; hTis work is licensed under a Creative Commons Attribution 4.0 International License. Nielsen, 2014; Gümüşderelioğlu et al., 2015; Toker et al., 2016; Dessordi et al., 2017; Doğan et al., 2017; Avşar Abdik et al., 2018) . Boron is quickly excreted in urine; thus, adverse effects on health are not expected with excess consumption (Price et al., 2012).

The goal of this work was to develop antimicrobial and biodegradable packaging materials from gelatin by incorporating boric acid (H3BO3) and its salts (disodium octaborate tetrahydrate: Na2B8O13-4H2O; sodium pentaborate: NaB5O15) as antimicrobial agents. To reach this goal, along with the antimicrobial properties, the chemical and physical properties of the developed films were investigated. To the authors’ best knowledge, this is the first study on developing boron-added gelatin films.

## 2. Materials and methods

### 2.1. Preparation of film-forming solutions (FFS) and antimicrobial gelatin films

Food-grade gelatin (Type B, 225 Bloom, from bovine skin) was supplied by Sigma-Aldrich (St. Lois, MO, USA). Boron derivatives (boric acid [BA], sodium pentaborate [SP], and disodium octaborate tetrahydrate [SO]) were provided by the National Boron Research Institute–BOREN (Ankara, Turkey) and Eti Maden (Ankara, Turkey). Glycerol was supplied by Merck (Darmstadt, Germany). Gelatin films were prepared using the solvent casting method.

Gelatin film-forming solutions were prepared by dispersing 3 g glycerol and 10 g powdered gelatin in 80 mL double distilled water at 50 °C with continuous stirring. Diefrent amounts of boron derivative (0.5g, 1.0 g, 1.5 g) were separately dissolved in 20 mL double-distilled water at 50 °C before being mixed with gelatin solution containing glycerol. The final mixture was stirred for 30 min. The resulting film-forming solution (20 mL) was poured onto plastic Petri plates and dried for 48 h at ambient temperature until the solvent was evaporated. Samples containing only gelatin and glycerol were used as the control. The dried gelatin films were peeled of the surface of the Petri plates to obtain the final film samples. The films containing 0.5 g of boron derivative, 1.0 g of boron derivative, and 1.5 g of boron derivative will be referred to as 5% (g/g gelatin), 10% (g/g gelatin), and 15% (g/g gelatin) respectively throughout the paper, based on the dry weight percentages (100 × mass of boron derivative/mass of gelatin).

### 2.2. pH and conductivity of film-forming solutions (FFSs)

The electrical conductivity and the pH of the FFSs were measured using a pH meter (PHM210, Radiometer Analytical SAS, Lyon, France) and a conductivity meter (CDM210, Radiometer Analytical SAS) at 50 °C.

### 2.3. Determination of antimicrobial properties of the films

The modified agar disc diffusion method was used to determine the antimicrobial activities of the developed gelatin films. Antibacterial and antifungal activities of the films with different boron derivatives were tested against a gram-positive bacteria (Staphylococcus aureus ATCC 6538), 2 gram-negative bacteria (Eshericia coli ATCC 10536 and Pseudomonas aeruginosa ATCC 15442), and 2 fungal isolates (Candida albicans ATCC 10231 and Aspergillus niger ATCC 16404). In the antimicrobial tests, tryptic soy agar (TSA) medium was used for the bacterial strains (E. coli, S. aureus, and P. aeruginosa) and potato dextrose agar (PDA) medium was used for the fungal isolates (C. albicans and A. niger).

To determine the antimicrobial activity, each of the developed films containing different boron derivatives at 3 different concentrations and a negative control film were aseptically cut into 1 × 1 cm square pieces. The aseptically prepared square film samples were placed on the surface of inoculated agar plates with a culture of the target indicator microorganism by using sterile tweezers. The inoculation loads were 10 8 cfu/mL and 104 cfu/mL for bacteria and fungi, respectively. The Petri dishes were divided into 4 sections and the film samples containing the same concentration of different boron derivatives (BA, SO, SP) and a negative control (film sample without boron derivatives) were placed on predefined sections.

Petri dishes were sealed with parafilm and incubated for 24 h at 25 °C for bacterial strains and for 48 h at 25 °C for fungal strains. Antimicrobial activities of the films were evaluated by measuring the inhibition zone area (colonyfree area) which had developed around the film squares with a digital caliper (Mitutoyo Corp, Tokyo, Japan). When an inhibition zone was not observed around a film sample, it was assumed that the sample did not have an inhibitory effect on the target microorganism. The antimicrobial tests were performed in triplicate for each sample.

### 2.4. Infrared spectroscopy

The Fourier-transform infrared (FTIR) spectrum of the developed gelatin films were recorded by scanning the film samples at wavelengths ranging from 4000 to 600 cm–1 in an infrared spectrometer (FT-IR Nicolet iZ10, eThrmo Scientific, Waltham, MA, USA).

### 2.5. Determination of the gelling and melting temperatures (points)

Dynamic viscoelasticity measurements were carried out using a controlled strain rheometer (Kinexus Malvern Instruments Ltd, Malvern, UK), and the rheological data were obtained from the instrument’s software (rSpace for Kinexus). All rheological measurements were performed in duplicate. Dynamic measurements were carried out using cup and bob geometry (CC25) to determine changes in gelling and melting temperatures of the gelatin samples containing different boron derivatives at different concentrations. The linear viscoelastic regions (LVR) of samples were measured to choose a strain value that would assure an intact network structure for all samples. An amplitude sweep test was performed for each sample at a constant frequency of 2 Hz and at 25 °C with increasing shear strain from 0.1% to 1000%. Four percent shear strain was chosen for the following oscillation tests. Temperature ramp tests were carried out at 4% shear strain and a constant frequency of 2 Hz. The crossover temperatures in the cooling cycle (from 40 °C to 18 °C at a rate of 1 °C/ min) and in the heating cycle (from 18 °C to 40 °C at a rate of 1 °C/min) were taken as the gelling temperatures of the FFS and the melting temperatures of the gelatin gels, respectively.

### 2.6. Mechanical properties of the films

Tensile strength (TS) of the films was measured according to the ASTM-D882 standard test method using a texture analyzer (TA.XTplus, Stable Micro Systems, Surrey, UK) with 5 kg load cell. The gap between tensile grips (A/MTG) was set to 50 mm. Film specimens (50 × 20 mm) of each formulation were clamped between tensile grips and each sample was pulled apart at a crosshead speed of 0.5 mm/s until it was torn. Measurements were done in triplicate for each sample. Films were conditioned (in a 50% RH chamber) for 48 h before analysis. TS was calculated by dividing the peak force by the cross-sectional area of the film (Force/thickness × 20 mm). The thickness of the films was measured with a digital caliper (Mitutoyo Corp, Tokyo, Japan). Thickness measurements were done in triplicate for each sample.

### 2.7. Morphology

Surface morphology of the gelatin film samples were examined with a scanning electron microscope (SEM) (EVO 40 series, Carl Zeiss, Oberkochen, Germany). Before the SEM imaging, film samples had been kept in a desiccator for 24 h. The surfaces of the films were coated with gold at 12–13 nanometers (BAL-TEC SCD 005 Sputter Coater, BAL-TEC GmbH, Schalksmühle, Germany) to enable sample imaging for SEM.

### 2.8. Statistical analysis

Microbiological and tensile strength measurements were replicated three times for each type of film. Statistical analyses were conducted with the analysis of variance (ANOVA) procedure in SPSS 20 Software (SPSS Inc., Chicago, IL, USA). Tukey’s test (P < 0.05) was used to detect differences among the mean values.

## 3. Results and discussion

Homogeneous and clear films with completely dissolved boron derivatives were achieved even at the highest concentration of the derivative used (15%, g/g gelatin). All films showed a characteristic yellowish color.

### 3.1. pH and conductivity of film forming solutions

Gelation of proteins can be altered by the addition of salt or a change in pH due to changes in the electrostatic interactions among chains. The isoelectric point of Type B gelatin is around 5.0 (Djagny et al., 2001; Gómez-Guillén et al., 2011) ; the pH of the gelatin FFSs prepared in this work was found to vary between 5.20 and 7.55 (Table 1). While the addition of BA did not change the pH value of the gelatin solution (around 5.20), the addition of borates increased the solution pH. The higher the concentration of the borates was, the higher were the pH values. Conductivity measurements showed that addition of boron derivatives has a similar effect on the conductivity as on pH. The 15% SO containing FFS was found to have the highest pH value and conductivity. The increase in solution pH and conductivity shows that both SO and SP act as alkaline salts in the gelatin solution. In order to understand whether the changes in pH and conductivity interfere with the gelation mechanism and the properties of the resulting gel, FTIR spectra, and melting/gelling points were determined and mechanical studies were conducted, as discussed in Sections 3.3, 3.4, and 3.5.

**Table 1:** pH and conductivity values of gelatin film-forming solutions with and without boron incorporation.

	Antimicrobial agent concentration(g/ g gelatin)	pH	Conductivity (mS/cm)
Control	-	5.23 ± 0.02	2.16 ± 0.01
Boric acid	5%	5.21 ± 0.01	1.86 ± 0.01
	10%	5.20 ± 0.01	2.01 ± 0.02
	15%	5.25 ± 0.02	2.25 ± 0.02
Disodium octaborate	5%	6.85 ± 0.00	2.75 ± 0.01
	10%	7.42 ± 0.01	3.94 ± 0.02
	15%	7.55 ± 0.01	4.69 ± 0.01
Sodium pentaborate	5%	6.58 ± 0.02	2.74 ± 0.02
	10%	7.23 ± 0.02	3.30 ± 0.01
	15%	7.42 ± 0.01	3.90 ± 0.02

### 3.2. Antimicrobial properties of the films

**Figure 1:**
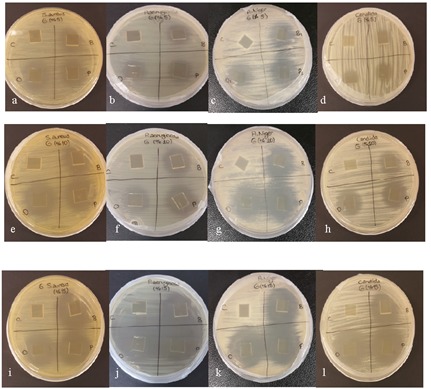
Representative visuals of inhibition zones of gelatin gels with 5% (g/g gelatin) (a,b,c,d), 10% (e,f,g,h), and 15% (i, j,k,l) boron derivative (control [C], boric acid[B], sodium pentaborate [P], disodium octaborate [O]) against S. aureus (a,e,i), P. aeruginosa (b,f,j), A. niger (c,g,k), and C. albicans (d,h,l).

The size and representative images of the inhibition zones of the gelatin films are given in Table 2 and Figure 1, respectively. The results show that gelatin films containing different boron derivatives showed inhibitory effect on S. aureus, P. aeruginosa , and A. niger for all film formulations. However, no inhibition effect was observed against E. coli. Growth of C. albicans was inhibited for all gelatin films except the ones containing boron derivatives at the lowest concentration tested (5%, g/g gelatin). At the same concentrations, boron derivatives inhibited the growth of S. aureus similarly. Increasing the concentration of the antimicrobial agent from 5% (g/g gelatin) to 10% (g/g gelatin) increased the inhibition zones; however, further increasing it to 15% (g/g gelatin) did not result in a significant difference in the inhibition of S. aureus. For P. aeruginosa, the inhibition zones formed by the films containing 5% SO (g/g gelatin) and 5% SP (g/g gelatin) were significantly higher than the films containing 5% BA (g/g gelatin). At 10% and 15% (g/g gelatin) concentrations, the inhibition effects of antimicrobial agents against P. aeruginosa were similar to each other. Increasing the concentration of BA from 5% (g/g gelatin) to 10% (g/g gelatin) and from 10% (g/g gelatin) to 15% (g/g gelatin) significantly increased the inhibition zones.

**Table 2:** 

Concentration (g/g gelatin)	Antimicrobial agent	Inhibition zones (mm) §
Concentration (g/g gelatin)	Antimicrobial agent	S. aureus	P. aeruginosa	E. coli	A. niger	C. albicans
0%	--	0.00	0.00	0.00	0.00	0.00m
5%	Boric acid	17.61 ± 1.29a	14.41 ± 1.25	0.00	24.59 ± 2.38i	0.00m
	Sodium pentaborate	17.70 ± 0.65a	20.15 ± 0.93d,e,f	0.00	25.57 ± 1.44i,j	5.17 ± 7.31m
	Disodium octaborate	19.22 ± 1.84a	19.02 ± 1.05d,e	0.00	26.93 ± 1.11i,j,l	4.25 ± 6.01m
10%	Boric acid	23.54 ± 1.32b,c	19.53 ± 1.20e	0.00	29.57 ± 0.52 ,k	20.44 ± 1.95n
	Sodium pentaborate	22.53 ± 1.13b	20.15 ± 0.93d,e,f	0.00	29.40 ± 1.49j,k	21.05 ± 0.89n
	Disodium octaborate	23.80 ± 0.69b,c	19.02 ± 1.05d,e	0.00	31.18 ± 0.58k,l	21.09 ± 1.22n
15%	Boric acid	26.80 ± 0.58c	24.79 ± 1.36f,g	5.04 ±7.13	32.17 ± 1.28k	23.62 ± 2.0n
	Sodium pentaborate	25.90 ± 0.18b,c	23.91 ± 1.28f,g	0.00	33.56 ± 1.00k	22.86 ± 3.11n
	Disodium octaborate	26.79 ± 1.03c	25.52 ± 1.36g	3.37 ±4.77	33.39 ± 0.55k	24.60 ± 2.68n

§ The mean values with the same letter within the same column are not significantly different (P > 0.05).

On the other hand, for SO, a significant increase in the inhibition zones was observed only when the concentration was increased from 10% (g/g gelatin) to 15% (g/g gelatin). In the case of E. coli, only small inhibition zones appeared around the films containing 15% BA (g/g gelatin) and 15% SO (g/g gelatin). For this reason, no statistical analysis was performed for E. coli inhibition. At the same concentrations, the boron derivatives inhibited the growth of A. niger similarly. Increasing BA concentration in the films from 5% (g/g gelatin) to 10% (g/g gelatin) increased the inhibitory effect on A. niger. However, further increase in the concentration of BA did not have a significant effect on inhibition. The inhibitory effect of SO and SP on A. niger did not change with concentration. While the gelatin films containing 5% (g/g gelatin) boron derivative did not show a significant inhibitory effect on C. albicans, films containing 10% (g/g gelatin) or 15% (g/g gelatin) antimicrobial agent had a significant antifungal effect on C. albicans. The results show that for the tested microorganisms, adding 10% (g/g gelatin) boron derivative to the gelatin films decreased cell viability significantly, except for E. coli (Figure 1). Increasing the concentration further to 15% (g/g gelatin), resulted in a significant change in the inhibition zones only for P. aeruginosa.

The inhibitory zones achieved in this work with incorporation of boron derivatives against G(+) bacteria, molds, and yeasts are promising compared to those of previous work in the literature (Clarke, 2016; Cozmuta et al., 2015; Kanmani et al., 2014a; Shankar et al., 2015) . However, one drawback of the present work is that the a e i b c k h l boron added gelatin gels did not show antimicrobial activity against E. coli.

### 3.3. Infrared spectroscopy

**Figure 2:**
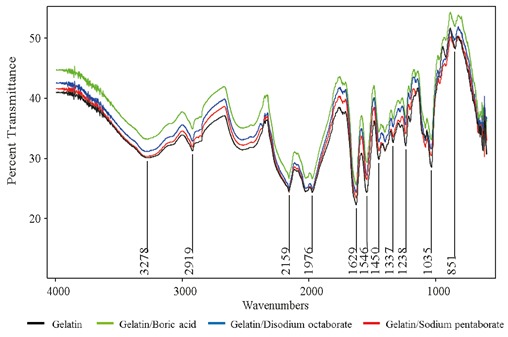
FTIR spectra of gelatin and gelatin films incorporating boron derivative.

FTIR analysis was used to characterize the changes induced by incorporation of boron derivatives into gelatin film matrix by distinguishing the IR bands and vibrational shifts related to boron derivative–gelatin interactions. Figure 2 shows the FTIR spectra of the gelatin and antimicrobial gelatin films. The characteristic absorption peaks appeared at 1629 cm–1, 1546 cm–1, and 1238 cm–1 which corresponds to C = O stretching (amide-I), N-H stretching (amide-II), and C-N and N-H stretching (amide-III), respectively. The peak at 1629 cm–1 indicates the frequency of carbonyl (C = O) stretching/hydrogen bonding coupled with COO. The characteristic peak at 2922 cm –1 corresponds to C-H stretching. All peaks observed on the FTIR spectrum of gelatin film and gelatin-based films with boron derivatives were similar except for peak heights, showing that there is no chemical bond formation between gelatin and added boron derivatives.

### 3.4. Determination of gelling and melting points

Random coil-helix reversion is the accepted gelation mechanism of gelatin. When a heated gelatin solution 8 7 2 3 with a concentration higher than 0.5% is cooled to its gelling temperature, flexible and disordered coils of gelatin associate into triple helices to form thermoreversible gels. These helices are stabilized in the junction zones by hydrogen bonds (Ramachandran and Reddi, 1976; Bigi et al., 2004) . If the gelatin gel is heated above its melting temperature again, the gel will melt because of the dissociation of the triple helices (Nur Hanani et al., 2014) . Ionic bonds and hydrophobic interactions also contribute to the gel formation (Kanmani and Rhim, 2014b; Pang et al., 2014) . The difference in gelling/melting temperatures of gelatin may result from the original collagen source, concentration, and molecular weight (Ferry and Eldridge, 1949; Veis, 1964; Clark and Ross-Murphy, 1987; Gilsenan and Ross-Murphy, 2000) . Increasing the number of physical/chemical interactions/bonds between chains will also result in higher melting temperatures. It has been reported that the effect of concentration and the type of the added salt on protein stability and gelling behavior is very specific (Von Hippel and Wong, 1962; Sarabia et al., 2000) . Moreover, the acting mechanisms might be varied, such as changes in levorotation, competition for water to hydrate, direct ion binding to the backbone, or indirect effect on protein folding due to interactions with structurally bound water (Harrington and Von Hippel, 1961; Asghar and Hendrickson, 1982; Elysée-Collen and Lencki, 1996) . For this reason, temperature ramp tests were conducted to investigate the effects of BA and its salts on the gelling temperature of FFSs and the melting temperature of corresponding gelatin gels. Table 3 shows the gelling/ melting points of the gelatin samples studied. It can be concluded that the addition of boron derivatives did not substantially alter the gelling temperature of gelatin FFSs or the melting temperature of the corresponding gelatin gels. The gelation mechanism mostly depends on the hydrogen bonds within the triple helical structures, which lead to the formation and stabilization of junction zones. It is known that when the number of helical structures decreases, the melting temperature decreases (Gilsenan and Ross-Murphy, 2000) . That there is no change in the melting temperature of gelatin gels with the addition of boron derivatives shows that incorporation of boron derivatives into the gelatin solutions does not interfere with the coil-helix transition, and hence the network formation. FTIR results also support this finding, since there are no additional chemical bonds occurring in the network to increase the melting temperature due to the incorporation of boron derivatives.

**Table 3:** Gelling (TG) points of gelatin FFSs and melting (TM) points of gelatin gels with different formulations.

	Control	Boric acid	Disodium octaborate	Sodium pentaborate
Concentration (g/g gelatin)	0%	5%	10%	15%	0.44%	10%	15%	0.44%	10%	15%
TG	22.7 °C± 0.2	22.3 °C± 0.1	22.2 °C± 0.1	21.4 °C± 0.2	22.3 °C± 0.1	21.8 °C± 0.1	21.4 °C± 0.1	22.3 °C± 0.2	21.9 °C± 0.1	21.4 °C± 0.2
TM	30.9 °C± 0.1	30.7 °C± 0.2	30.5 °C± 0.1	30.4 °C± 0.2	30.9 °C± 0.1	31.1 °C± 0.1	30.5 °C± 0.1	31.1 °C± 0.3	30.5 °C± 0.2	30.5 °C± 0.1

### 3.5. Mechanical properties of the films

Mechanical properties of biopolymer packaging systems are important in assessing their degree of resistance. In order to understand the effect of boron addition on the mechanical properties of the gelatin films, the tensile strength of the films was measured. Compared to the control film, incorporation of boron derivatives mostly enhanced the tensile strength (Table 4). While the average peak force value of the control film was 9851 g, values of the average peak forces ranged from 11,375 g to 17,172 g depending on the concentration and the type of boron derivative. In polymer-based systems, tensile strength increases when the ordered structure and the crystalline packing of the polymer chains increase (Bradbury and Martin, 1952) , since the linear molecular orientation (Agn/gtimgeilcartoinb)ial agent concentration Tensile strength§ (MPa) Film thickness (mm) § The mean values with the same superscript letters are not significantly different (P > 0.05). increases the resistance of the polymer system against the tensile force. Gelatin is a partially crystalline polymer at ambient temperature (Sobral and Habitante, 2001) . The crystalline phase is associated with the triple helix structure, which is important in gel formation (Duconseille et al., 2017) . Thus, any structural changes or interactions that would promote the ordered structure would increase the tensile strength. Results show that the tensile strength values of SO incorporated gels are significantly higher than the tensile strength of the control at all concentrations. hTis may be attributed to the increased order in the spatial arrangement and association of the gelatin chains due to the screened charges by the addition of SO, which acts like an alkaline salt in the gelatin solution as evidenced by the increased pH and higher conductivity values (Table 1). Once dissolved, SO provides more ions compared to SP, which might result in more salt effect on the protein chains than that provided by SP. The tensile strength values of gelatin films produced in this study are found to be comparable to and in some cases even higher than the tensile strengths of gelatin films reported in previous studies (Carvalho and Grosso, 2004; Carvalho and Grosso, 2006; Bae et al., 2009; Wang et al., 2009; Guerrero et al., 2011; Nur Hanani et al., 2012a; Nùñez-Flores et al., 2013) .

**Table 4:** The effect of boron derivatives on the tensile strength and the film thickness of gelatin films.

	Antimicrobial agent concentration (g/g gelatin)	Tensile strength§ (MPa)	Film thickness (mm)
Control	-	18.58 ± 2.79a	0.26 ± 0.01
Boric acid	5%	25.31 ± 0.63a,b	0.24 ± 0.02
	10%	23.20 ± 2.00b,c	0.29 ± 0.01
	15%	24.35 ± 0.86b,c	0.28 ± 0.01
Disodium octaborate	5%	30.27 ± 1.54c,d	0.26 ± 0.01
	10%	30.07 ± 1.27d	0.28 ± 0.00
	15%	26.71 ± 2.88c,d	0.28 ± 0.00
Sodium pentaborate	5%	21.45 ± 0.64a,b	0.26 ± 0.01
	10%	24.98 ± 1.15b,c	0.25 ± 0.01
	15%	22.00 ± 0.92a,b	0.26 ± 0.00

§ The mean values with the same superscript letters are not significantly different (P > 0.05).

Moreover, the tensile strength values of all samples are higher than the tensile strength values of LDPE films, which were reported as 13 MPa by Arvanitoyannis and Biliarderis (1999) and between 8.6 MPa and 17.3 MPa by Andreuccetti et al. (2009) . The thickness of the gelatin films with and without boron derivatives varied between the range of 0.24 and 0.29 mm (Table 4).

### 3.6 Morphology

**Figure 3:**
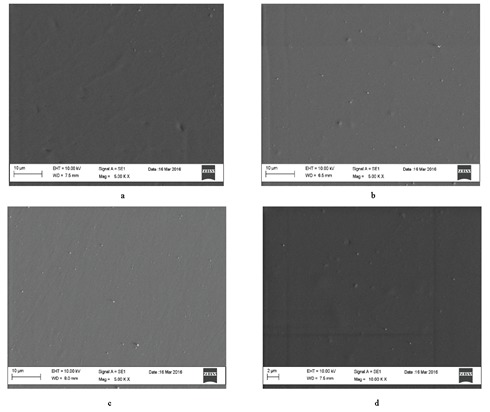
Scanning electron micrograph of surface of (a) negative control gelatin film, and scanning electron micrographs of surfaces of gelatin films containing the highest amounts of boron derivatives, (b) 15% boric acid (g/g gelatin), (c) 15% disodium octaborate (g/g gelatin), (d) 15% sodium pentaborate (g/g gelatin).

Scanning electron micrographs of the surface of the gelatin films can be seen in Figure 3. The gelatin films showed a smooth surface structure. Antimicrobial films showed a similar structure to the control films.

## 4. Conclusion

Gelatin is one of the most common biopolymers used for biodegradable packaging/film studies. In this work, we developed clear, homogenous, antimicrobial gelatin films with boron derivatives that have antibacterial effect against gram-positive bacteria S. aureus and gram-negative bacteria P. aeruginosa, and antifungal effect against fungi A. niger and C. albicans. The results show that adding 10% boron derivative (g/g gelatin) to the gelatin films decreased the viability of the tested strains significantly except for E. coli. Increasing concentration further to 15% (g/g gelatin) resulted in a significant change in the inhibition zones only for P. aeruginosa.

Furthermore, the tensile strength values of gels incorporating disodium octaborate were found to be significantly higher than the tensile strength of the control at all concentrations. No change was observed in the gelling temperatures of the gelatin FFSs or in the melting temperatures of the gelatin gels with the addition of boron derivatives, which suggests that boric acid and its salts do not interfere with the coil-helix transition, and hence the network formation. This finding is also in good agreement with the FTIR results that showed no bond formation between boron derivatives and gelatin chains to increase the melting temperature. The morphology of antimicrobial films with added boron was found to be similar to the morphology of the control sample.

These results suggest that when antimicrobial, physical, and chemical properties are considered together, gelatin films containing 10% or 15% disodium octaborate (g/g gelatin) might be good candidates for biodegradable antimicrobial packaging materials. In conclusion, boron derivatives oefr the advantage of being cheap, safe, and eefctive agents for developing antimicrobial gelatin films with good tensile properties.
